# Projected Estimates of African American Medical Graduates of Closed Historically Black Medical Schools

**DOI:** 10.1001/jamanetworkopen.2020.15220

**Published:** 2020-08-20

**Authors:** Kendall M. Campbell, Irma Corral, Jhojana L. Infante Linares, Dmitry Tumin

**Affiliations:** 1 Research Group for Underrepresented Minorities in Academic Medicine, Division of Academic Affairs, Brody School of Medicine, East Carolina University, Greenville, North Carolina; 2 Division of Behavioral Medicine, Department of Psychiatry and Behavioral Medicine, East Carolina University, Greenville, North Carolina; 3 Office Data Analysis and Strategy, Brody School of Medicine, East Carolina University, Greenville, North Carolina; 4 Division of Academic Affairs, Department of Pediatrics, Brody School of Medicine, East Carolina University, Greenville, North Carolina

## Abstract

**Importance:**

There continue to be low numbers of underrepresented minorities, including African Americans, in academic medicine. Historically Black medical colleges and universities are major sources of training for medical school graduates who are African American or who belong to other underrepresented minority groups. Several historically Black medical schools were closed during the period surrounding the 1910 Flexner report. The implications of these school closures with regard to the number of African American medical school graduates have not been fully examined.

**Objective:**

To examine the consequences associated with the closure of historically Black medical schools for the number of African American medical school graduates.

**Design, Setting, and Participants:**

This observational economic evaluation used steady expansion and rapid expansion models to estimate the consequences associated with the closure of historically Black medical schools for the number of African American medical school graduates. The numbers of graduates from 13 historically Black medical schools that are now closed were obtained through historical records. Data on historically Black medical schools that are currently open were obtained from school-specific reports and reports published by the Association of American Medical Colleges. The study focused on projected estimates of outcomes from the hypothetical continued operation and expansion of 5 closed historically Black medical schools that were included in the Flexner report: Flint Medical College of New Orleans University, Knoxville Medical College, Leonard Medical School of Shaw University, Louisville National Medical College, and the University of West Tennessee College of Medicine and Surgery–Memphis.

**Main Outcomes and Measures:**

The main outcome was the estimate of the number of African American students who would have graduated from historically Black medical schools that were closed during the period surrounding the 1910 Flexner report.

**Results:**

Among the 5 historically Black medical schools that were closed, the estimated mean number of graduates per year was 5.27 students at Flint Medical College, 2.60 students at Knoxville Medical College, 11.06 students at Leonard Medical School, 4.17 students at Louisville National Medical College, and 6.74 students at the University of West Tennessee. If the 5 closed historically Black medical schools had remained open, the steady expansion and rapid expansion models indicated that these schools might have collectively provided training to an additional 27 773 graduates and 35 315 graduates, respectively, between their year of closure and 2019. In the analysis of Leonard Medical School and the University of West Tennessee only, the steady expansion and rapid expansion models indicated that these 2 schools would have provided training to an additional 10 587 graduates and 13 403 graduates, respectively, between their year of closure and 2019. An extrapolation based on the racial and ethnic self-identification of current graduates of historically Black medical schools indicated that if these closed schools had remained open, the number of graduating African American physicians might have increased by 355 individuals (29%) in 2019 alone.

**Conclusions and Relevance:**

To increase the number of African American medical school graduates, consideration should be given to creating medical education programs at historically Black colleges and universities. Such programs may start with small enrollment but could have positive consequences for the diversity of the physician workforce.

## Key Points

**Question:**

What are the projected estimates of the number of African American students who would have graduated from historically Black medical schools that were closed during the period surrounding the publication of the 1910 Flexner report?

**Findings:**

In this economic evaluation of 13 historically Black medical schools that were closed and 4 historically Black medical schools that remained open after the 1910 Flexner report, an extrapolation based on data from the medical schools that remained open indicated that 5 of the closed medical schools might have collectively provided training to an additional 35 315 graduates by 2019. If these 5 closed schools had remained open, they could have produced a 29% increase in the number of graduating African American physicians in 2019 alone.

**Meaning:**

The study’s findings suggest that consideration should be given to the creation of medical education programs at historically Black colleges and universities in an effort to increase the number of African American graduates from medical schools and the number of African American physicians in the workforce.

## Teaser

This economic evaluation uses data from historically Black medical schools that were closed during the period surrounding the publication of the 1910 Flexner report to examine the implications of these school closures for the number of African American medical school graduates and physicians.

## Introduction

Increasing the number of physicians from underrepresented minority groups, including those who are African American or Black, has been an objective of many medical schools. Although an increase in representation has occurred, the number of African American medical faculty remains limited.^1^ The shortage of African American faculty can have adverse consequences for the recruitment and retention of minority learners in medical schools. Many medical schools currently use holistic admissions processes, pipeline and outreach programs, and other initiatives to increase the number of students from underrepresented minority groups.^2,3^ However, even with an increase in the number of schools that are addressing issues regarding poor and underserved communities, health disparities, and social factors associated with health,^4^ the increase in the number of African American students, especially male students, remains slow.^5^ Although the opening of additional medical schools may have created additional opportunities for students from minority groups to begin their medical educations, to our knowledge, none of the nearly 30 new medical schools that have opened since 2000 were located at historically Black colleges or universities.^6^ Coupled with the increasing number of graduate medical education programs and the ongoing initiatives to increase the number of African Americans attending predominantly White institutions, the creation of new medical schools at historically Black colleges and universities may help to increase the numbers of African American medical school graduates and have positive consequences for the physician workforce.

Historically Black colleges and universities can be an important resource for recruiting students from underrepresented groups to medical programs.^7^ These colleges and universities are incubators for young minority talent and often produce positive academic outcomes despite limited funding, negative media images, a history of marginalization, fewer faculty members, and inadequate facilities.^8,9,10^ Partnerships between predominantly White institutions and historically Black colleges and universities can be symbiotic, with a sharing of assets that benefits faculty and students.^11^ Although fewer in number, historically Black medical schools graduate proportionately more students from underrepresented minority groups than do medical schools at predominantly White institutions.^12^ Increased numbers of graduates from minority groups can translate into a more supportive learning environment, in which there is less effort needed for relationship building because of the similarities in culture and background between faculty and students.^10^ Black race-concordant faculty and student environments can promote the personal and social development of students and foster academic gains in writing skills, technology, science, and the arts.^13^ Four historically Black medical schools are currently in operation: Meharry Medical College (Meharry), Howard University College of Medicine (Howard), Morehouse School of Medicine (Morehouse), and Charles R. Drew University of Medicine and Science (CDU). However, 13 other historically Black medical schools were operating in the late 1800s to early 1900s and were closed owing to low enrollment and limited resources.^4^ These medical programs operated in 7 states in the eastern part of the US, from Louisiana to Pennsylvania, and collectively graduated more than 700 students (Table 1).

**Table 1.** Graduates of Closed Historically Black Medical Schools

School	Dates open	Years open, No.	Graduates, No.	Estimated graduates per year, mean
Included in extrapolation analysis				
Flint Medical College of New Orleans University, Louisiana	1889-1911	22	116^a^^,^^b^	5.27
Knoxville Medical College, Tennessee^c^	1900-1910	10	26	2.60
Leonard Medical School of Shaw University, North Carolina^d^	1882-1918	36	398^b^	11.06
Louisville National Medical College, Kentucky	1888-1912	24	>100^e^	4.17
University of West Tennessee College of Medicine and Surgery–Jackson^e^	1900-1907	7	16	2.29
University of West Tennessee College of Medicine and Surgery–Memphis	1907-1923	16	155^a^	6.74
Not included in extrapolation analysis				
Chattanooga National Medical College, Tennessee	1899-1904	5	16^a^	3.20
Hannibal Medical College, Tennessee^f^	1889-1896	7	5^a^	0.71
Knoxville College Medical Department, Tennessee	1895-1900	5	2^a^	0.40
Lincoln University Medical Department, Pennsylvania^g^	1870-1874	4	0	0
Medico-Chirurgical and Theological College of Christ's Institution, Maryland	1900-1908	8	ND	NA
State University Medical Department, Kentucky^h^	1899-1903	4	ND	NA
Straight University Medical Department, Louisiana^i^	1873-1874	1	0	0

Abbreviations: NA, not applicable; ND, no data.

^a^ Total number of graduates obtained from Harley.^14^

^b^ Exact number of graduates unknown in the final years before closure.

^c^ Changed from Presbyterian to independent affiliation.

^d^ No MD degrees awarded after 1912. Total number of graduates obtained from the Medical Department of Shaw University.^15^

^e^ School moved its campus to Memphis in 1907.

^f^ No MD degrees awarded after 1892.

^g^ A total of 6 students graduated, but none completed MD degree.

^h^ Merged with Louisville National Medical College.

^i^ A total of 2 students graduated, but none completed MD degree.

An important factor in the closure of these medical programs was the Flexner report on medical education, which was published in 1910. This report emphasized the importance of education in basic sciences as a prerequisite and a complement to clinical experience in the training of physicians.^16^ With regard to the historically Black medical schools operating at the time, the Flexner report recommended that only the Howard and Meharry programs remain operational, as Flexner believed these schools alone had the potential to meet the new requirements for curricula, faculty, and facilities.^5,6^ Although historically Black medical schools faced other challenges during this period, including limited funding and facilities, student financial hardship, and the cumulative disadvantage of discrimination and segregation in premedical education,^14,17^ the Flexner report represented a turning point. After the publication of this report, all but 2 of the historically Black medical schools, including several that were thriving at the turn of the century (as indicated by enrollment and graduation patterns),^18^ closed. In the present study, outcomes of historically Black medical schools that are currently open were used to extrapolate projected workforce estimates of the number of African Americans who would have graduated with medical degrees if the schools that were closed after the Flexner report had been preserved and expanded.

## Methods

Historical records were used to identify the number of graduates from 13 historically Black medical schools that are now closed. Sources of the data included Association of American Medical Colleges (AAMC) reports spanning the 2009 to 2010 through 2018 to 2019 graduation years,^19,20^ school-specific reports of the number of graduates,^15^ and previous research describing historically Black medical schools that were operating before the publication of the Flexner report. Schools that were closed before the Flexner report was published (ie, before 1910) were excluded, as those schools had few graduates and their closure was likely inevitable during that period. Therefore, the study focused on hypothetical outcomes of the continued operation and expansion of 5 historically Black medical schools that were included in the Flexner report: Flint Medical College of New Orleans University (Flint), Knoxville Medical College (Knoxville), Leonard Medical School of Shaw University (Leonard), Louisville National Medical College (Louisville), and the University of West Tennessee College of Medicine and Surgery–Memphis (West Tennessee).^14^ Among the schools that eventually closed, only Leonard and West Tennessee remained open in the immediate period after the Flexner report and were still operational as of 1912^8^; therefore, we examined a second scenario that was limited to the continued operation and expansion of these 2 schools. This study followed the Consolidated Health Economic Evaluation Reporting Standards (CHEERS) reporting guideline for economic evaluation studies.

To estimate the projected increase in the number of graduates from these schools, the last year of each school’s operation was identified. Because the actual number of graduates in the last year of operation was unknown for 3 of the 5 schools (Flint, Leonard, and Louisville), we assumed that the baseline number of graduates per year was equivalent to the mean number of annual graduates during the school’s existence. Two models were explored to calculate patterns in the number of graduates. First, a steady expansion model was applied based on data from Howard and Meharry, the 2 schools that have remained in continuous operation since the Flexner report. Although Howard initially received greater financial support than Meharry and operated its own hospital (which Meharry did not), both schools had similar numbers of African American graduates who entered the physician workforce. For example, between the 1940s and 1960s, 50% of African American physicians received training at Howard, and 40% of African American physicians received training at Meharry; today, the schools continue to have graduating classes of similar sizes.^17,21^ Therefore, we extrapolated the rate of expansion based on the combined number of graduates from these 2 schools between 1930 (when the combined number of graduates stabilized at approximately 100 students)^18^ and 2019 (when the schools graduated a total of 181 students, for an expansion rate of approximately 0.9 graduates per year).^19,20^

Second, a rapid expansion model was applied based on data from Morehouse, at which the number of 4-year graduates increased from 20 students in 1985 to 73 students in 2019. Using complete annual data on the number of Morehouse graduates during this period,^19,20,22^ a linear regression model was applied to calculate an annual expansion rate of 1.3 graduates (95% CI, 1.1-1.5 graduates). In both models, it was assumed that each school would reach a maximum annual number of 100 graduates (in comparison, the number of Howard graduates in 2010-2019 ranged from 97 to 111 graduates per year, and the number of Meharry graduates ranged from 86 to 104 graduates per year)^19,20^; the rapid expansion model implied that this theoretical maximum would be reached sooner than it would in the steady expansion model. For each model, the hypothetical expansion rate was calculated from the date of each school’s closure until 2019, and the rate was then multiplied by the number of years to determine the number of additional physicians who would have received training during this time.

To provide a conservative estimate of the consequences for the diversity of the contemporary physician workforce if these schools had remained open, 2019 data from the AAMC were used to extrapolate the proportion of graduates who would have identified as African American from each school that might have remained open. This proportion was based on the percentage of African American graduates among physicians graduating from Howard, Meharry, and Morehouse in 2019 (202 of 285 total graduates [71%]), which was the most current year for which data were available.^21^ The number of African American physicians who would have graduated from the schools had they remained open was then compared with the total number of African American physicians who graduated from existing allopathic medical schools in 2019 (1238 graduates).

Data from CDU were included for comparison purposes, as this historically Black university operates a joint medical education program with the University of California, Los Angeles (UCLA). Students complete foundational coursework at the UCLA campus and clinical training at the CDU campus.^23^ The joint program enrolled its first class of 21 students in 1981 and continues to operate today, with cohorts of approximately 24 students each year.^24,25^ Because of the distinct structure of the CDU program as a joint initiative with UCLA, we did not use CDU data when formulating possible trajectories of expansion for historically Black medical schools that were operational before the publication of the Flexner report.

## Results

Among the 5 historically Black medical schools that were closed, the estimated mean number of graduates per year was 5.27 students at Flint, 2.60 students at Knoxville, 11.06 students at Leonard, 4.17 students at Louisville, and 6.74 students at West Tennessee. The specific numbers of graduates of closed historically Black medical schools are shown in Table 1. Among the historically Black medical schools that were currently open, the number of African American graduates from 2015 to 2019 comprised 36 students from CDU, 316 students from Howard, 339 students from Meharry, and 207 students from Morehouse. The specific numbers of graduates from these schools are summarized in Table 2.

**Table 2.** African American Enrolled Students and Graduates of Currently Open Historically Black Medical Schools

School	No.				Male to female ratio of 2018-2019 graduates^d^
	Enrolled students in 2019-2020^a^	Graduates in 2019-2020^b^	Graduates in 2018-2019^b^	Graduates in 2015-2019^c^	
Charles R. Drew University of Medicine and Science, California^e^	72	22	4	36	1.2:1.0
Howard University School of Medicine, District of Columbia	316	108	67	314	1.0:1.0
Meharry Medical College, Tennessee	335	104	82	339	0.7:1.0
Morehouse School of Medicine, Georgia	260	73	53	207	0.8:1.0

Abbreviation: AAMC, Association of American Medical Colleges.

^a^ Data obtained from the AAMC.^26^

^b^ Data obtained from the AAMC.^21^

^c^ Total graduates by US medical school and race/ethnicity from 2013 to 2014 through 2018 to 2019 obtained from the AAMC.

^d^ Data obtained from the AAMC.^20^

^e^ Data from the joint program of the AAMC and the University of California, Los Angeles, provided by the Charles R. Drew University Office of Institutional Research.

The simulated expansion in the number of historically Black medical school graduates at the closed schools is summarized in Table 3, with comparisons of projected estimates from the steady expansion model vs the rapid expansion model and from the continued operation of all 5 medical schools that were open at the time of the Flexner report vs the continued operation of Leonard and West Tennessee only. In the steady expansion model compared with the rapid expansion model, the 5 closed medical schools would have collectively provided training to an additional 27 773 graduates and 35 315 graduates, respectively, between their year of closure and 2019. In the analysis of Leonard and West Tennessee only, the steady expansion and rapid expansion models indicated that these 2 schools would have provided training to an additional 10 587 graduates and 13 403 graduates, respectively, between their year of closure and 2019. Regardless of the expansion model used, all schools would have reached our hypothetical maximum of 100 graduates per year by 2019. Therefore, in the 5-school scenario, the number of African American medical school graduates in 2019 might have increased by 355 graduates (29%); in the 2-school scenario, this number might have increased by 142 graduates (11%).

**Table 3.** Projected Estimates of Additional Graduates From Closed Historically Black Medical Schools

School	Estimated graduates per year, mean	Years between school closure and 2019, No.	Additional projected graduates from year of school closure to 2019, No.	
			Steady expansion model^a^	Rapid expansion model^b^
Flint Medical College of New Orleans University, Louisiana	5.27	108	5862	7396
Knoxville Medical College, Tennessee	2.60	109	5678	7300
Leonard Medical School of Shaw University, North Carolina	11.06	101	5750	7102
Louisville National Medical College, Kentucky	4.17^c^	107	5646	7216
University of West Tennessee College of Medicine and Surgery–Memphis, Tennessee	6.74	96	4837	6301
Total projected graduates				
All 5 schools	NA	NA	27 773	35 315
Leonard Medical School and University of West Tennessee only	NA	NA	10 587	13 403

Abbreviation: NA, not applicable.

^a^ Assumes an expansion rate of 0.9 graduates per year to a maximum of 100 graduates per year.

^b^ Assumes an expansion rate of 1.3 graduates per year to a maximum of 100 graduates per year.

^c^ Mean number of graduates based on the lower limit of the known total number of graduates (Table 1).

## Discussion

During the first 2 decades of the 20th century, the number of medical schools that provided training to African American students rapidly decreased, a development associated in part with the 1910 Flexner report, which recommended that, among the historically Black medical schools, only the Howard and Meharry programs should remain operational.^14^ The present study aimed to examine a long-standing question regarding the ways in which the continued operation of additional historically Black medical schools would have changed the physician workforce in the US. Using data from the 2 historically Black medical schools that remained open (Howard and Meharry) and data from the recent expansion of a new medical school at a historically Black college (Morehouse), we estimated that between 10 000 and 30 000 additional physicians could have received training from the historically Black medical schools that were closed after the publication of the Flexner report. Given the persistent challenges of increasing the representation of medical students and graduates from underrepresented minority groups at predominantly White institutions, which currently operate all but 4 of the medical schools in the US, these results suggest that investing in the creation of additional medical schools at historically Black colleges and universities may have long-lasting implications for the size and diversity of the physician workforce in the US.

Historically Black medical schools graduate a disproportionately high number of African American physicians in the US, despite receiving less financial support than medical schools at predominantly White institutions.^27,28^ Before the 1960s, historically Black medical schools graduated nearly all of the African American physicians who received training in the US and, as recently as 2010, historically Black medical schools provided training to 1 in 5 Black medical school graduates in the US.^17,29^ Although not all US medical schools were formerly segregated, as of 1968, Black students represented only 2% of medical student enrollment. In response to these data, the AAMC announced in 1970 that its goal was to increase the enrollment of Black medical students to 12%.^17^ Despite slow progress over the subsequent 50 years, this percentage of representation has not yet been achieved. The proportion of African American physicians was 6% in 1991 and 7% in 1997, and it had not reached 8% as of 2019.^30^ Although various initiatives at the medical schools of predominantly White institutions were undertaken to support the recruitment and graduation of African American students, this lack of expansion suggests that increasing the availability of medical education at historically Black colleges and universities, a strategy last implemented in 1981 with the founding of the Morehouse School of Medicine, could be an important step toward meeting the AAMC’s 1970 goal.

Among the principal challenges of historically Black medical schools before the Flexner report was securing the facilities and funding to enable their continued operation.^14^ This challenge was compounded by ongoing segregation within the medical profession and the educational system in general.^18,31^ The AAMC requirement that medical school matriculants have a 4-year high school diploma, for example, was associated with a decrease in historically Black medical school enrollment that started as early as 1906, 4 years before the publication of the Flexner report.^18^ Nevertheless, in the first decade of the 20th century, several historically Black medical schools across the eastern US persevered and expanded. The continued operation of additional schools among this number might have had both quantitative (as our calculations indicated) and qualitative consequences for diversity in the medical profession and the status of health disparities in the US.^32^

The early (pre–World War II) implications of preserving additional historically Black medical schools are difficult to characterize given the overall changes in medical education during this period.^18^ Enrollment and graduation numbers would have remained relatively low even if schools had undertaken a program of rapid expansion after the Flexner report. Yet, the continued operation of these programs may have had important implications for the number of physicians serving African American communities and provided training to a generation of physicians who might have inspired and mentored many other students from underrepresented minority groups to pursue a career in medicine. The continued operation of additional historically Black medical schools and the potential for associated increases in the number of African American physicians during this time might have also factored into the efforts of the National Medical Association to overturn the segregationist practices and policies of the American Medical Association.^33^

Extrapolation of the continued operation of additional historically Black medical schools to more recent years reveals the extent to which their continued operation could have had positive consequences for the contemporary physician workforce. Based on the most conservative assumptions in this study, more than 10 000 additional physicians would have received training if only 2 additional historically Black medical schools had remained open and expanded at the same rates as Howard and Meharry; as of 2019, this expansion rate would have represented an 11% increase in the number of African American physicians graduating from US medical schools each year. In comparison, the actual increase in the number of African American graduates of US medical schools over the last 5 years (2015-2019) was 1%.^34^ Based on the assumption that 5, rather than 2, additional historically Black medical schools had remained in operation, the increase in the number of African American graduates could have been as high as 29% in 2019. In this scenario, African Americans would still have accounted for only 8% of graduating physicians in 2019, which is lower than the 13% needed to achieve equal representation compared with the general US population.^35^ Over the long term, however, the positive consequences of operating additional medical schools at historically Black colleges and universities may help to close this gap. Because many graduates of historically Black medical schools attain leadership positions in medical schools at predominantly White institutions, the operation of additional historically Black medical schools may also address challenges associated with minority student recruitment and graduation rates at predominantly White institutions by increasing the diversity of the faculty and administration at these schools.^36^

### Limitations

This study has several limitations. As with other studies that have estimated projected outcomes from the hypothetical continued operation of historically Black medical schools that were closed in the early 20th century, our analysis was constrained by limited data and incomplete information about the contemporary opportunities and challenges encountered by the schools’ administrators.^18^ In addition, variable and incomplete data on year-specific enrollment and graduation rates prevented us from performing more complex mathematical modeling (eg, incorporating nonlinearity in the projected rates of expansion). As observed in data from the historically Black medical schools operating today, expansion occurs in stages rather than in a linear manner. Using retrospective analysis to estimate when a school might have, for instance, added 10 more spots to its enrollment class was beyond the scope of available data. Furthermore, our estimates of projected increases in the number of graduates focused on the pattern of the total number of physicians who received training at each school but not specifically on the number of African American physicians who received training. Reliable data on the racial and ethnic self-identification of medical students before the 1970s are limited, and we concluded that jointly modeling changes in the number of graduates and the proportion of African American graduates would have required excessive assumptions given the data available.

The potential consequences of the continued operation of additional historically Black medical schools remain a matter of conjecture and are dependent on the distinct circumstances of each institution.^14^ What is more likely, however, is that the closure of these schools, combined with other changes in medical education, curtailed the number of new African American physicians, helping to create a disparity that persisted across generations and continues to have implications for the medical workforce more than a century later.

## Conclusions

Our study projected estimates of the number of African American physicians who would have graduated from closed historically Black medical schools if those schools had remained open. Even with the use of conservative assumptions, the continued operation of additional historically Black medical schools after the publication of the Flexner report in 1910 could have changed the course of American medicine, both quantitatively, through a substantial increase in the diversity of the physician workforce, and qualitatively, through an increase in the number of mentors and role models who might have encouraged many African American students to pursue a career in medicine. Furthermore, because physicians and students from underrepresented minority groups graduating from historically Black medical schools have disproportionately pursued clinical practice, research, and advocacy that target the needs of medically underserved communities,^28,37^ the continued operation of additional historically Black medical schools might have produced substantial increases in the medical profession’s capacity to address health disparities. Reflecting on this alternative history of historically Black medical schools after the Flexner report points the way toward initiatives to support the creation and expansion of medical schools at historically Black colleges and universities. With recent changes in medical education, including the rapid increase of online education and the potential for more cost-effective instruction during the preclinical years, the barriers to the formation of new medical schools may be lower now than they have been for several years.^38^ These schools might start with small enrollment but could have long-lasting implications for the diversity of the physician workforce.
